# Information-Seeking Behavior for COVID-19 Boosters in China: A Cross-Sectional Survey

**DOI:** 10.3390/vaccines11020323

**Published:** 2023-01-31

**Authors:** Xiaoshan Austin Li, Qiwei Luna Wu, Katharine Hubbard, Jooyun Hwang, Lingzi Zhong

**Affiliations:** 1 Department of Communication, Faculty of Humanities and Social Sciences, BNU-HKBU United International College, 2000 Jintong Rd., Zhuhai 519087, China; 2 School of Communication, Levin College of Public Affairs and Education, Cleveland State University, 2121 Euclid Ave., MU 233, Cleveland, OH 44115, USA; 3 Mass Communication Department, Sam Houston State University, 1905 University Ave, Huntsville, TX 77340, USA; 4 Department of Journalism, Public Relations, and Advertising, Soongsil University, 369 Sangdo-Ro, Dongjak-Gu, Seoul 06978, Republic of Korea; 5 Department of Communication and Huntsman Cancer Institute, University of Utah, 2000, Circle of Hope, Room 4513, Salt Lake City, UT 84112, USA

**Keywords:** vaccination, COVID-19 boosters, PRISM, risk information seeking, China, knowledge insufficiency, affect

## Abstract

As China launches its second COVID-19 booster campaign and races to bring new vaccine technologies to protect against severe COVID-19 infections, there is limited research on how Chinese residents search for vaccine-related information. This study examined the factors influencing Chinese residents’ information-seeking behaviors regarding COVID-19 boosters with a sample of 616 respondents with a mean age of 31.53 from a research panel. Structural equation modeling was used to report factors that influenced respondents’ seeking intent. The results indicated that seeking-related subjective norms (*β* = −0.55, *p* < 0.001), negative affect (*β* = 0.08, *p* < 0.05), positive affect (*β* = 0.18, *p* < 0.001), and perceived knowledge insufficiency (*β* = 0.10, *p* < 0.001) are strong predictors of one’s seeking intent. We also discovered that there was an inverse relationship between risk perception and positive affect (*β* = −0.55, *p* < 0.001) and between negative and positive affect (*β* = −0.19, *p* < 0.01), while all measurements were either directly or indirectly related to information-seeking intent. A few more indirect but important relationships were also included in our discussion. In conclusion, the present study helps understand what motivates Chinese residents to seek COVID-19 booster information when limited information is available.

## 1. Introduction

While China’s COVID-19 cases are surging to record highs, Chinese officials are determined to trim back a raft of the zero-COVID infrastructure to embark on a new journey to live with COVID-19. However, the number of confirmed positive cases has spiked exponentially since the radical departure from the onerous COVID restrictions [1]. An adviser on China’s COVID-19 task team warned the public that more than 60% of the 1.4 billion population might be infected [2]. Although, as of December 14, 2022, 1.27 billion Chinese have been fully vaccinated, resulting in a coverage of over 90% of the population [3], and mounting concern and anxiety have rippled through the country. As a counter measurement, the National Health Commission (NHC) touts the launch of its second COVID-19 vaccine booster campaign to expedite the administration of booster doses, especially for people over 60 years old.

Currently, Chinese authorities are racing to bring more COVID-19 booster doses that endorse new technologies. They recently approved two inhalable vaccines for emergency use [4], but little is known about the effectiveness of the new inhaled vaccines, despite being studied worldwide [4]. Equally, while China is close to approving ArCoV, potentially the first Chinese-made mRNA vaccine [5], the emergence of these new technologies may introduce further sources of uncertainty in addition to current concerns about potential adverse effects that have been identified. Given the heightened importance of staying current with vaccinations, the public is likely to engage in the purposeful gathering of information to combat uncertainty [6,7], which, in turn, may lead to vaccine acceptance or rejection. Numerous studies have investigated people’s acceptance of vaccination, e.g., [6,7,8,9]. During this challenging time, understanding how Chinese people seek information is just as important as knowing what promotes their acceptance of booster doses. However, to our knowledge, little work has been carried out to understand what motivates people to seek information in the context of receiving COVID-19 booster shots.

The current study deploys a robust framework—the planned risk information seeking model (PRISM)—to depict underlying motivators of COVID-19 booster dose information-seeking intent among Chinese people. PRISM was initially examined in a general health context [10] and later has expanded to diverse contexts, e.g., cancer risks [10,11,12], seismicity [13,14], and political risks [15]. More precisely, PRISM suggests risk-related seeking behaviors are motivated by whether individuals perceive themselves as capable of searching for information (i.e., perceived behavioral control) and if they sense a gap between their current and ideal knowledge (i.e., knowledge insufficiency). At the same time, social pressure (i.e., subjective norms), perceived risks, and evoked emotions (e.g., negative and positive affects) all contribute to one’s information-seeking behaviors—particularly in the context of risk. Therefore, the PRISM model is a potential candidate to serve as the starting point for comprehending risk-information-seeking intent in China’s context of COVID-19 booster doses.

The present study attempts to fill the gap by examining factors that spark vaccine-related information seeking when Chinese people decide whether to take COVID-19 booster doses. Under uncertainty, seeking information is the first step to self-education and reconciling with it. As the first study to explore this matter, we expect this study to set a baseline for future scholarly work. A better understanding of information-seeking intent can offer policymakers and the public a better picture of booster dose uptake.

## 2. Materials and Methods

### 2.1. Study Design

We conducted a cross-sectional survey between November 13 and 23, 2022 in China. The survey was conducted online using panel participants from Wen Juan Xing, a leading Chinese survey platform with three million registered and representative members (e.g., [9]). The research team developed a Chinese questionnaire using the original English PRISM [10] measures. We evaluated the questionnaire using a pilot study.

To begin with, a Chinese research assistant translated the PRISM questionnaire into Chinese. We solicited feedback from 10 native Chinese speakers who held doctoral degrees in different fields but were not involved in the current study. Upon receiving their feedback, multiple revisions were made to target the accuracy and language fluency of the questionnaire. We validated the Chinese PRISM questionnaire with 72 participants from Wen Juan Xing’s research panel. Cronbach’s alpha values (greater than 0.70) suggested that results satisfied internal reliabilities.

Wen Juan Xing recruited participants to complete the finalized survey if they were (1) living in China and (2) 18 years old and above. Participants were allowed to finish the survey at their own pace. Respondents, on average, took about 25 minutes to complete the survey. Out of 735 returned questionnaires, 119 responses were excluded from the final analysis if they completed the survey in an unrealistically short time (e.g., five mins) or failed the attention check question. As a result, the survey’s completion rate was 83.8%, comparable to other online survey studies [16].

### 2.2. Measures

The study primarily deployed the measurements from the original planned risk information-seeking model (PRISM) model, as it is a well-cited framework to examine one’s information-seeking intent across various contexts. We noted the cited work in correspondence to the constructs for benefit perception and positive affect. We also include the complete questionnaire in the Appendix A.

*Attitude Toward Seeking*. Respondents rated on five semantic scales to indicate “to what extent you feel that seeking information about the risks and benefits associated with COVID-19 booster doses is ….” The 5-point semantic differential pairs included: bad (1)/good (5), harmful (1)/beneficial (5), unhelpful (1)/helpful (5), foolish (1)/wise (5), unproductive (1)/productive (5). We averaged the items to create a composite, with higher scores representing a more positive attitude toward information seeking (*M* = 4.15. SD = 0.59. Cronbach’s α = 0.74).

*Seeking-related Subjective Norms*. Participants rated their agreement (1 = strongly disagree, 5 = strongly agree) on four items (e.g., “Most of my family whose opinions I value expect me to seek information about the risks and benefits posed by receiving COVID-19 booster doses”). The items were averaged to create a composite, with higher scores representing higher normative expectancy for booster-related information seeking (*M* = 3.88, *SD* = 0.684, Cronbach’s α = 0.80).

*Seeking-related Seeking Control*. To measure the construct, participants rated their agreement (1 = strongly disagree, 5 = strongly agree) on four items (e.g., “I know where to look for information about the risks and benefits of receiving COVID-19 booster shots”). We created a composite by averaging the four items; a higher composite score reflects better control (*M* = 3.98, *SD* = 0.62, Cronbach’s α = 0.74).

*Risk Perceptions.* Perceptions of risk are often captured as the perceived likelihood of the risk occurring and the perceived seriousness of the risk if it were to occur. We measured risk likelihood with four questions, “how likely is it that you will be impacted by the potential risks posed by COVID-19 booster shots,” “if you were impacted by the potential risks posed by COVID-19 booster shots, how serious would that impact be,” “how likely is it that society will be impacted by the potential risks posed by COVID-19 booster doses,” and “if society were impacted by the potential risks posed by COVID-19 booster doses, how serious would that impact be?”. Response options were on a 5-point Likert scale ranging from “1 = not at all likely/serious” to “5 = extremely likely/serious”. The items were averaged to create a composite, with higher scores representing higher risk perceptions (*M* = 3.12, *SD* = 1.26, Cronbach’s α = 0.90).

*Benefit Perceptions.* Unlike risk perception, where sets of established measurements are at our disposal, benefit perception measures seem arbitrary across contexts. We modified six items based on a few scholarly works examining mask wearing and acceptance of the COVID-19 vaccine [17,18,19] to ask respondents about their perceptions of benefit. Participants were instructed to rate their agreement (1 = strongly disagree, 5 = strongly agree) on the items (e.g., “COVID-19 booster doses will decrease my chance of getting COVID-19 or its complications”). We averaged the items to create a composite, with higher scores reflecting higher benefit perceptions (*M* = 4.22, *SD* = 0.59, Cronbach’s α = 0.83).

*Negative Responses.* We intended to stay consistent with previous measurements of negative affective responses but also expanded them to include positive affect. For negative affective responses, we based our measures on Witte’s (1992) fear appeals but modified them to five items: “When I think about COVID booster shots, I get… frightened,” “…tense,” “…nervous,” “…anxious,” and “…uncomfortable.” Participants rated their agreement with these statements on a 7-point scale (1 = strongly disagree, 7 = strongly agree). Items were averaged to create a composite, with higher scores representing more negative responses toward the booster shots (*M* = 2.7, *SD* = 1.28, Cronbach’s α = 0.91).

*Positive affect* is “a state of pleasurable engagement with the environment that elicits feelings, such as happiness, joy, excitement, enthusiasm, and contentment” [20]. We assessed positive affect using a 7-point Likert scale that ranged from “1 = strongly disagree” to “7 = strongly agree.” Respondents were prompted: “When I think about COVID-19 booster doses, I get… hopeful,” “…happy,” “…enthusiastic,” “…relieved,” and “…confident.” The five items were averaged to create a composite, with higher scores representing more positive responses toward the booster shots (*M* = 5.51, *SD* = 0.94, Cronbach’s α = 0.87).

*Perceived Knowledge.* Consistent with Kahlor’s [10] original measurement, we used a single item to measure participants’ current knowledge of COVID-19 booster doses on a scale of 0 to 100, where zero indicates a person knows nothing about the subject while 100 means they know everything they could possibly know (*M* = 68.35, *SD* = 17.38).

*Perceived Risk Knowledge Sufficiency Threshold.* This concept describes the discrepancy between a person’s current knowledge level and their perceived level of knowledge. To measure this difference, we asked, “How much do you need to know about the risks and benefits posed by COVID-19 booster doses?” As with perceived knowledge, this item was measured on a scale of 0 to 100 (“0 = nothing” to “100 = all there is to know.”; *M* = 67.02, *SD* = 19.62).

*Seeking Intent*. Information-seeking intent was measured with four items on a Likert-type scale ranging from “1 = strongly disagree” to “5 = strongly agree” (e.g., “I will try to seek information about the risks and benefits posed by COVID-19 booster doses in the next six months,” “I will look for information about the risks and benefits posed by COVID-19 booster doses in the next six months,” Kahlor, 2010). Items were averaged to create a composite, with higher values reflecting stronger intentions to seek information related to the COVID-19 booster shots (*M* = 3.98, *SD* = 0.7, Cronbach’s α = 0.85).

### 2.3. Data Analysis

Firstly, respondents’ demographic characteristics and vaccination histories were demonstrated using frequencies and percentages.

**Figure 1:**
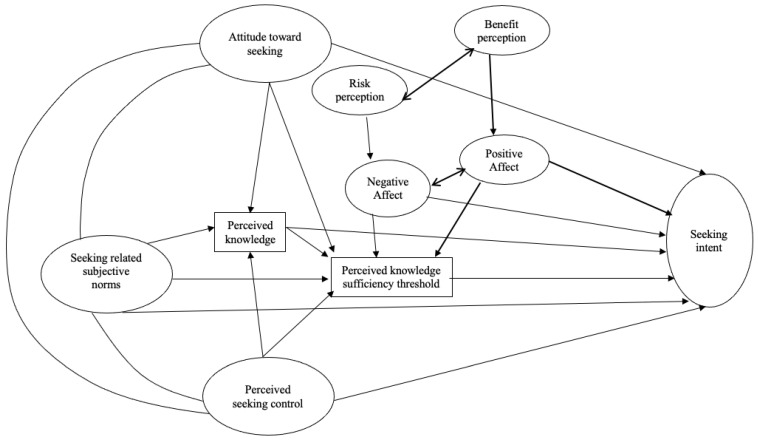
Demonstrations of the planned risk information seeking model with benefit perception and positive affect.

Then, we turned our attention to the model that includes exogenous variables, such as attitude, norm, control, risk perception, and benefit perception, along with endogenous variables including perceived knowledge, perceived knowledge sufficiency threshold, negative and positive affect, and intention. Non-directional paths were drawn among attitude, norms, control, as well as between risk perception and benefit perception, and negative and positive affect. Perceived knowledge was regressed on attitude, norms, and control. Perceived knowledge sufficiency threshold was regressed on perceived knowledge, attitude, norms, and control. Positive affect was regressed on benefit perception; negative affect was regressed on risk perception. Intention was regressed on all other study variables (Figure 1).

To assess the extent to which the proposed factors influence Chinese people’s seeking intent regarding COVID-19 vaccines, we lean into conventional statistical figures, including path coefficient (*β*) and significance level (*p*-value), to draw conclusions.

Additionally, traditional structural equation modeling analysis reports a model’s overall fitness. Therefore, we also examined the model fit and the proposed factors. Indicators of model fit included the chi-square goodness-of-fit [21], the comparative fit index (CFI; values greater than 0.90), Tucker–Lewis index (TLI; values greater than 0.90), root mean square error approximation (RMSEA; values lower than 0.08), and standardized root mean residual (SRMR; values lower than 0.08) [21,22]. Maximum likelihood with robust standard errors (MLR) was employed as an estimation method. The data analysis was carried out and Mplus 8.3 was used to run the structural equation modeling.

## 3. Results

### 3.1. Participants

Of all participants, 52.4% were female (*N* = 323). The mean age was 31.5 years old (*SD* = 6.5). Most participants have bachelor’s degrees and currently live in a metropolitan area while working full time (See Table 1).

### 3.2. Vaccination History

Approximately 96% of the respondents were fully vaccinated or received at least one booster dose. Since the respondents are (1) primarily stationed in China and (2) foreign vaccines are not available in the Chinese market, about 82% of them injected Chinese brands developed by three major Chinese vaccine companies: Sinovac, BIBP, and CanSino. Interestingly, 168 of the respondents received vaccines other than domestically produced ones, which implied they previously were outside of mainland China (Table 2).

### 3.3. Model Fit and Relationships

The measurement and structural models of the PRISM with benefit and positive affective responses presented very good model fits (Table 3). Therefore, we conclude that PRISM is an adequate model to explain the motivators behind Chinese people’s seeking intent in the context of COVID-19 booster shots.

**Figure 2:**
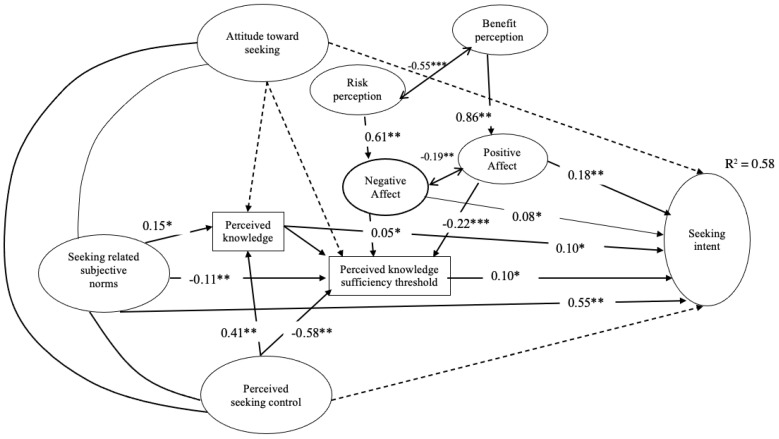
Structural equation model of PRISM with benefit and positive affect. Dotted lines represent insignificant paths. * *p* < 0.05. ** *p* < 0.01. *** *p* < 0.001.

Furthermore, we closely examined the influencing relationships among variables. Specifically, the relationship between benefit perception and positive affect (*β* = 0.86, *p* < 0.001) was stronger than the relationship between risk perception and negative affect (*β* = 0.61, *p* < 0.001). In fact, changes in benefit perception are likely to induce a more pronounced change in positive affect than in risk perception and negative affect. Similarly, the relationship between positive affect (*β* = 0.18, *p* < 0.001) and seeking intent and that between negative affect and seeking intent (*β* = 0.08, *p* < 0.05) were similar, but the bond between positive affect and intention was still more substantial than that between the latter one. Note that while negative affect was positively related to perceived knowledge insufficiency (*β* = 0.01, *p* < 0.05), the relationship between positive affect and knowledge insufficiency was significant but negatively related (*β* = −0.22, *p* < 0.01). Moreover, risk perception and benefit perception were inversely related (*β* = −0.55, *p* < 0.001), as was negative and positive affect (*β* = −0.19, *p* < 0.01). Figure 2 includes all paths and their significances. For a complete summary of relationships and their significance levels, please refer to Table 4.

Insignificant relationships (bolded in Table 4) surfaced between attitude toward seeking and seeking intent (*β* = −0.01, *p* = 0.84); attitude toward seeking and perceived knowledge (*β* = 0.05, *p* = 0.55); attitude toward seeking and knowledge insufficiency (*β* = 0.001, *p* = 0.47); and perceived seeking control and seeking intent (*β* = 0.10, *p* = 0.12). Although the relationship between seeking-related subjective norms and perceived knowledge insufficiency threshold was statistically significant, it turned out to be a negative one (*β* = −0.001, *p* < 0.01).

## 4. Discussion

The outbreak of SARS-CoV-2 has gauged an unprecedented amount of attention from scholarly communities across disciplines. Numerous studies have investigated people’s acceptance of preventive behaviors, such as vaccination injections e.g., [6,7] during the pandemic and booster doses as a continuum e.g., [8,9]. Given that the virus continuously mutates, people still bear uncertainty; research has generally concluded that uncertainty is a natural force of information seeking because the public needs to bring closure to this unwanted tension [23]. Acknowledging that the COVID-19 pandemic is a defining experience for many, we turned our attention to what motivates people to seek information to deal with uncertainty in such a risky context.

The present study dissects motivational factors behind Chinese people’s information-seeking intent of COVID-19 booster doses. Not only are there an insufficient number of studies on understanding public information seeking during the pandemic, but even fewer focus on China, where policies and pandemic preventative measures are notably different from the rest of the world.

### 4.1. Theoretical and Practical Implications

Our results suggest that seeking-related subjective norms, negative affect, positive affect, and perceived knowledge insufficiency are strong predictors of one’s seeking intent. In addition, PRISM, along with benefit perceptions and positive affect, surface as robust predictors for exploring motivations behind people’s information-seeking intent in the study context. The model presented a good fit and explained nearly 60% of the variance in seeking intent.

Central to the present study was the influence of benefit perception and positive affect on other latent variables in PRISM. In reality, people do not analyze risks separately from benefits when making their judgments about a perceived risk [24,25,26,27]. The present study confirmed the assumption that benefit and risk perceptions are inversely related to each other, which might indicate a reciprocal cause. An increase in risk perception could cause a decrease in benefit perception and vice versa.

Likewise, while risk and benefit perceptions were inversely correlated, negative and positive affect were the byproducts of these two perceptions. Our results indicated that negative and positive affect were inversely related as well. For example, if Chinese people feel happy about receiving COVID-19 booster shots, they should feel less anxious about the side effects. As mentioned, people are susceptible to affect heuristics, which are “positive and negative tags associated with the representations consciously or unconsciously” [28] when making judgments and decisions. In other words, affect often serves as a shortcut for people when making decisions. Moreover, positive affect was strongly but negatively related to the knowledge insufficiency threshold. That is, people tend to believe they have adequate knowledge when they feel happy or relieved about COVID-19 booster shots. The downside is that people may overlook potential risks or knowledge gaps when in a positive mood.

In addition to the statistically significant relationships, the unsupported ones offer opportunities for future explorations. For one, attitude toward seeking was not a strong predictor of seeking intent. In this study, attitude was a weak predictor in seeking intent, perceived knowledge, and knowledge insufficiency threshold. Past studies, by and large, have suggested the strong predictive power of attitude in the three relationships [11,12,29]. Presumably, this weak relationship could be a result of the study context: China’s zero-COVID policy restrictively imposed lockdowns and quarantines even if there was only one confirmed case. In other words, people lived in a “COVID-free” bubble. To elaborate, strict zero-COVID policies required confirmed cases to be as close to zero as possible. Therefore, it signaled a complete COVID-19-free environment as long as the official headcount was zero and there were no lockdowns. When a low number of cases were reported, it did not escalate much tension to urge the unvaccinated group to be vaccinated because the zero-COVID strategies protected them. Also, fully vaccinated or boosted people were not exempted from lockdowns and quarantines if they were identified as close contacts, lived, or worked in the same area. That is, people did not receive any incentives for being vaccinated. With no sense of urgency and no reward for receiving vaccines, attitude towards booster shots can generally be mild or indifferent.

Furthermore, a vast majority of people had already received two doses of Chinese vaccines by the time we collected the data. Additionally, the evaluation of the risks and benefits of booster shots could have been indifferent or weak because the situation may have been less relevant to people due to zero-COVID policies at the time of data collection. However, it is possible that people’s attitudes toward seeking information related to booster doses could change with changing policies.

Apart from the unexpectedly poor performance of attitude, the relationship between perceived seeking control and seeking intent was positively related but insignificant (*β* = 0.11, *p* = 0.11). We were not surprised by the insignificance as it has occurred in a few studies across different contexts [11,12,13]. On the one hand, as Willoughby and Myrick [12] suggested, the insignificant relationship could result from study contexts and population. On the other hand, as of December 2020, 9.89 million Chinese had access to the Internet. Roughly 30% seek medical and healthcare information on the Internet [30]. Perhaps the prevalence of the Internet and the familiarity of internet searches have made perceived seeking control less relevant in this context. The unsupported relationship may direct scholars to investigate the influences that the Internet places on one’s perceived information-seeking control. 

A more surprising relationship surfaced between seeking-related subjective norms and perceived knowledge insufficiency. Theoretically, social norms can be either injunctive (i.e., approval of behavior) or descriptive (i.e., what others do) [31]. From the point of view of injunctive norms, supposedly, when people feel pressured to conform to the norms (e.g., seeking COVID-19 booster information) they are compelled to report a lower need for knowledge. The negative relationship in this study indicated a complex situation. Since we decided to simultaneously prompt people on the risks and benefits posed by COVID-19 boosters, we compromised the possibility of distinguishing between whether they felt pressured to seek risks or benefits and the information that they already had or needed. This was also a dilemma Kahlor and her team [13] faced in their study of understanding people’s information-seeking intent in carbon capture and storage. Our decision to keep risks and benefits together was based on the study context in which teasing them apart did not reflect reality, and for the sake of keeping the survey within a reasonable length.

Admittedly, our emphasis on the injunctive norms might have overshadowed the prominence of descriptive norms in the current study context. China is a country that ranks high in collectivism, and people from such a cultural context tend to value in-group members’ opinions [32,33]. Therefore, it is seemingly possible that descriptive norms may work in favor of one’s seeking intent if people they relate to exercise such behavior. In practice, framing a message that features what others do could potentially promote one’s seeking intent. For example, a public service announcement that features an elderly person who is going from vaccine-hesitant to vaccine-accepting may boost confidence among Chinese elderly who are currently under-vaccinated. Future work should consider adding descriptive norms to expand the score of perceived social norms, which may help better explain the relationship between norms and knowledge insufficiency.

### 4.2. Limitations and Future Research

Despite the contributions the study has made, our study comes with a few limitations. First, because the zero-COVID policy created an unprecedented situation that people experienced for three years, the data collected during the zero-COVID period could not capture what is currently happening. A longitudinal study is needed to compare individuals’ information-seeking intent during policy changes. Secondly, the present study is focused on seeking intentions rather than actual behaviors. Research over time has reached an agreement that social-cognitive models such as the theory of planned behavior (TPB) are better models to predict intentions than actual behaviors e.g., [34,35,36], which scholars refer to as the “intention-behavior gap” [37,38]. As Ajzen [39] suggested, we agreed that including other variables might help close the gap.

Furthermore, our analysis depends solely on participants’ conscious self-assessment of their current conditions. Self-reported measurements tend to suffer from social desirability bias; respondents might be sensitive to portraying themselves as naïve or uninformed [40]. Additionally, we are not clear on how well individuals can accurately access or report their attitudes [41]; thus, attitudes or estimates of one’s knowledge may be inaccessible or skewed. To deal with this limitation, future research can rely on additional measurement methods to triangulate with direct self-report data, such as implicit attitudinal measures [42].

A further limitation of this study is that we worded many of our model constructs with wording that simultaneously invoked “risks and benefits” rather than setting them apart. It is possible that people may weigh risks and benefits differently, which thus impacts their attitudes towards the context and their perceived level of control related to seeking information from those sources. Future research needs to better tease these concepts apart.

Last but not least, we were inclusive of respondents’ vaccination histories. We purposely made this decision based on maximizing our understanding of Chinese people’s information-seeking intent. Additionally, we shared the same vision as the WHO [43], that the COVID-19 booster dose may become an annual injection. In other words, we consider receiving booster doses likely to be a repeated health behavior, as will seeking information about boosters. Therefore, we intended to use this study to establish a baseline understanding. Future studies can further segment respondents based on their boosters’ histories if histories related to boosters may play a role in explaining individuals’ seeking behavior.

## 5. Conclusions

Scholarly attention has been sparse concerning cognitive factors influencing the acceptance of COVID-19 booster doses, but people do not make decisions without information or knowledge. Thus, more work should be devoted to how people react to the presented information. Overall, the present work’s findings highlight the importance of dissecting how factors, such as perceived risks and benefits, affective responses, and knowledge insufficiency, could significantly influence the public’s intentions to seek information in complex risk situations. Including a full scale of positive emotions and perceived benefits suggests a new pathway for future research to focus on risk-related communication in troubling times.

## Figures and Tables

**Table 1:** Demographic Information.

	M (SD)	N (%)
**Age**	31.53 (6.59)	
**Sex**		
**Male**		293 (47.6)
**Female**		323 (52.4)
**Education**		
**General education**		2 (0.3)
**High school**		18 (2.9)
**Some college**		66 (10.7)
**Bachelor’s degree**		486 (78.9)
**Postgraduate degree**		44 (7.1)
**Location**		
**Northeast China**		16 (2.6)
**North China**		125 (20.3)
**Central China**		77 (12.5)
**East China**		201 (32.6)
**South China**		125 (20.3)
**Northwest China**		19 (3.1)
**Southwest China**		53 (8.6)
**Overseas**		0 (0)
**Area**		
**Rural**		18 (2.9)
**Municipal**		158 (25.6)
**Metropolitan**		413 (67)
**Suburb**		27 (4.4)
**Income**		
**≤** **5000**		98 (15.9)
**5000–10,000**		260 (42.2)
**10,000–15,000**		185 (30)
**≥** **15,000**		73 (11.9)
**Employment**		
**Unemployed/student/full-time caregiver**		54 (8.8)
**Part time**		12 (1.9)
**Full time**		546 (88.6)
**On leave**		1 (0.2)
**Retired**		3 (0.5)

**Table 2:** Vaccination History.

	N	%
**Vaccination**		
**None**	8	1.3
**Partial**	17	2.8
**Fully**	130	21.1
**Booster**	461	74.8
**Booster**		
**1**	213	34.6
**2**	26	4.2
**3**	210	34.1
**4**	12	1.9
**Reason**		
**Not convenient**	29	12.50%
**Not safe**	20	8.60%
**Lack instructions**	56	24.10%
**Don’t care**	33	14.20%
**Not efficient**	26	11.20%
**Location unclear**	52	22.40%
**Medical restriction**	11	4.70%
**Other**	5	2.20%
**Brand**		
**BIBO**	302	30.50%
**SinoVac**	457	46.10%
**CanSino**	55	5.50%
**Pfizer**	78	7.90%
**Moderna**	16	1.60%
**Janssen**	48	4.80%
AstraZeneca	26	2.60%
**Others**	9	0.90%

**Table 3:** Fit statistics for measurement model and structural model for PRISM with benefit and positive affective responses.

Model	X^2^	*df*	CFI	TLI	Rmsea [90% C.I.]	Srmr
Benefit–Positive affect						
Measurement model	933.98	532	0.946	0.941	0.037 [0.034, 0.040]	0.036
Structural model	1286.534	677	0.957	0.953	0.038 [0.035, 0.041]	0.053

**Table 4:** Relationship Coefficients (*β*) and Corresponding *p*-values.

Relationships	*β*	*p*-Value
Attitude toward seeking is positively related to information-seeking intent	−0.04	0.55
Seeking-related subjective norms are positively related to information-seeking intent	0.55	0.00
Perceived seeking control is positively related to information-seeking intent	0.10	0.12
Risk perception is positively related to affective (negative) risk response	0.61	0.00
Benefit perception is positively related to affective positive affective response	0.86	0.00
Affective (negative) risk response is positively related to perceived knowledge sufficiency threshold	0.05	0.02
Positive affective response is positively related to perceived knowledge sufficiency threshold	0.22	0.03
Affective (negative) risk response is positively related to information-seeking intent	0.08	0.05
Positive affective response is positively related to information-seeking intent	0.18	0.02
Attitude toward seeking is positively related to perceived knowledge	0.05	0.44
Seeking-related subjective norms are positively related to perceived knowledge	−0.15	0.01
Perceived seeking control is positively related to perceived knowledge	0.42	0.00
Attitude toward seeking is positively related to perceived knowledge sufficiency threshold	0.01	0.47
Seeking-related subjective norms are positively related to perceived knowledge sufficiency threshold	−0.001	0.01
Perceived seeking control is negatively related to perceived knowledge sufficiency threshold	−0.01	0.01
Perceived knowledge insufficiency is positively related to information-seeking intent	0.10	0.00

## Data Availability

The data presented in this study are available on request from the corresponding author. The data are not publicly available due to privacy issues.

## Appendix A. Study Questionnaire

	Using the following adjective scales, please indicate to what extent you feel that seeking information about the risks and benefits posed by receiving COVID booster shots is…
Attitude toward Seeking (1–5 scale)	1. Bad…………………………. Good 2. Harmful……………………. Beneficial
	3. Unhelpful ……………………Helpful
	4. Foolish …………………………………. Wise
	5. Unproductive ………………Productive
	Please read the following statements and indicate your level of agreement or disagreement. Circle one answer for each item. Scale 1–5, strongly disagree to strongly agree
Seeking-related Subjective Norms (1–5 scale)	1. Most of my close friends who are important to me think that I should seek information about the risks and benefits posed by receiving COVID-19 booster shots. 2. Most of my family whose opinions I value expect me to seek information about the risks and benefits posed by receiving COVID-19 booster shots. 3. Most of my family expects me to seek information about the risks and benefits posed by receiving COVID-19 booster shots.
	4. Most of my close friends expect me to seek information about the risks and benefits posed by receiving COVID-19 booster shots.
	Please read the following statements and indicate your level of agreement or disagreement. Scale 1–5, from strongly disagree to strongly agree.
Perceived Seeking Control (1–5 scale)	1. I know where to look for information about the risks and benefits of receiving COVID-19 booster shots.
	2. When it comes to finding information about the potential risks and benefits posed by receiving COVID-19 booster shots, I know what to do. 3. I can readily access information I need about the risks and benefits posed by receiving COVID-19 booster shots. 4. When it comes to information about the potential risks and benefits posed by receiving COVID-19 booster shots, I know how to separate fact from fiction.
Risk Perception (1–5 scale)	Please rate the overall level of risk posed to you by receiving COVID-19 booster shots. Use a scale of 1–5. 1 = not at all likely/serious, 5 = extremely likely/serious
	1. If you were impacted by the potential risks posed by COVID-19 booster shots, how serious would that impact be.
	2. How likely is it that you will be impacted by the potential risks posed by COVID-19 booster shots.
	3. How likely is it that society will be impacted by the potential risks posed by COVID-19 booster shots. 4. If society were impacted by the potential risks posed by COVID-19 booster doses, how serious would that impact be.
	Please read the following statements and indicate your level of agreement or disagreement. Scale 1–5, from strongly disagree/not at all beneficial to strongly agree/extremely beneficial.
Benefit Perception (1–5 scale)	1. I believe that COVID-19 booster shots could be an effective precautionary measure. 2. COVID-19 booster shots will decrease my chance of getting COVID-19 or its complications. 3. With the COVID-19 booster shots, I will collaborate to reduce the spread of the COVID-19 virus. 4. By taking the COVID-19 booster shots, I will help public authorities to combat the COVID-19 virus. 5. How likely is it that society will be impacted by the benefits posed by COVID-19 booster shots. 6. How much benefit do you think COVID-19 booster shots pose to you?
	The following statements describe my feelings about receiving booster shots. When I think about COVID booster shots, I get… (scale 1–7, from not at all to extremely)
Affect Responses (1–7 scale)	1. Hopeful 2. Happy 3. Enthusiastic 4. Relieved 5. Confident
	Please read the following statements and indicate your level of agreement or disagreement. Scale 1–5, from strongly disagree to strongly agree.
Information Seeking Intent (1–5 scale)	1. I will look for information about the risks and benefits posed by receiving COVID-19 booster shots in the next six months. 2. I intend to look for information related to the risks and benefits posed by receiving COVID-19 booster shots in the next six months. 3. I intend to find more information about the risks and benefits posed by receiving COVID-19 booster shots in the next six months. 4. I will try to seek information about the risks and benefits posed by receiving COVID-19 booster shots in the next six months. 5. In the next six months, I plan to seek information about the risks and benefits posed by receiving COVID-19 booster shots.
Perceived Knowledge	Rate your knowledge of the potential risks and benefits posed by receiving COVID booster shots on a scale of 0–100, where zero means knowing nothing about the topic and 100 means knowing everything you could possibly know about the potential risks and benefits.
Perceived Knowledge Insufficiency	Think of that same 0–100 scale again. This time, estimate how much knowledge you need to deal adequately with the potential risks/benefits posed by receiving COVID booster shots.
